# Translational Control Protein 80 Stimulates IRES-Mediated Translation of p53 mRNA in Response to DNA Damage

**DOI:** 10.1155/2015/708158

**Published:** 2015-07-26

**Authors:** Marie-Jo Halaby, Yan Li, Benjamin R. Harris, Shuxia Jiang, W. Keith Miskimins, Margot P. Cleary, Da-Qing Yang

**Affiliations:** ^1^The Hormel Institute, University of Minnesota, Austin, MN 55912, USA; ^2^Cancer Biology Research Center, Sanford Research/University of South Dakota, Sioux Falls, SD 57104, USA; ^3^The Masonic Cancer Center, University of Minnesota, Minneapolis, MN 55455, USA

## Abstract

Synthesis of the p53 tumor suppressor increases following DNA damage. This increase and subsequent activation of p53 are essential for the protection of normal cells against tumorigenesis. We previously discovered an internal ribosome entry site (IRES) that is located at the 5′-untranslated region (UTR) of p53 mRNA and found that the IRES activity increases following DNA damage. However, the mechanism underlying IRES-mediated p53 translation in response to DNA damage is still poorly understood. In this study, we discovered that translational control protein 80 (TCP80) has increased binding to the p53 mRNA *in vivo* following DNA damage. Overexpression of TCP80 also leads to increased p53 IRES activity in response to DNA damage. TCP80 has increased association with RNA helicase A (RHA) following DNA damage and overexpression of TCP80, along with RHA, leads to enhanced expression of p53. Moreover, we found that MCF-7 breast cancer cells with decreased expression of TCP80 and RHA exhibit defective p53 induction following DNA damage and diminished expression of its downstream target PUMA, a proapoptotic protein. Taken together, our discovery of the function of TCP80 and RHA in regulating p53 IRES and p53 induction following DNA damage provides a better understanding of the mechanisms that regulate IRES-mediated p53 translation in response to genotoxic stress.

## 1. Introduction

The tumor suppressor protein p53 inhibits cell transformation by stopping cell growth or triggering apoptosis. It is mutated in more than half of all human cancers, and the inactivation of the p53 pathway plays a major role in the process of oncogenesis [1]. Under unstressed conditions, p53 protein levels are usually low, and this protein exists in an inactive form. The level of p53 increases only when the cells are stressed or damaged [1, 2]. Induced p53 is then activated through multiple posttranslational modifications. The accumulation and activation of p53 allow it to function as a tumor suppressor. Activated p53 protein binds to specific target DNA sequences and stimulates transcription of a variety of downstream target genes. The upregulation of the proteins encoded by these genes results in cell growth arrest to maintain genetic integrity of the cell or apoptosis to eliminate the damaged cell.

Since elevated levels of p53 protein are known to be important in initiating the events leading to cell growth arrest or apoptosis after cellular stress [1, 2], regulation of p53 induction has been a major area of cancer research over the last three decades. Although it is known that p53 is stabilized and therefore accumulates in the cell after DNA damage, there is also clear evidence showing that an increase in p53 synthesis in response to DNA damage, such as ionizing radiation (IR) or ultraviolet (UV) irradiation, also contributes to increased p53 levels in the cell [2–5]. It was demonstrated that p53 biosynthesis increases rapidly in response to IR in mouse 3T3 cells, even after treating the cells with the transcription inhibitor actinomycin D [6]. Also, exposure to IR or etoposide was found to lead to an increase in the association of p53 mRNA with polysomes, which further suggests an increase in p53 translation [7, 8]. The mechanism underlying translational regulation of p53 induction via its 5′-UTR has started to emerge.

It is known that cap-dependent initiation of protein translation is used by the majority of mRNAs, since almost all eukaryotic mRNAs have an N^7^-methylguanosine cap structure at their 5′-ends [9]. eIF-4E is a translation initiation protein that binds to the cap structure. A translation repressor, eIF4E-binding protein 1 (4E-BP1, also called PHAS-I), inhibits cap-dependent translation by binding to eIF-4E [10, 11]. In quiescent cells, 4E-BP1 is hypophosphorylated and binds tightly to eIF-4E. Binding between 4E-BP1 and eIF-4E blocks the assembly of the eIF-4F protein translation initiation complex. Addition of growth hormones, such as insulin and IGF-I, induces phosphorylation of 4E-BP1 and causes the release of eIF-4E from 4E-BP1, which facilitates the translation of capped mRNA by making eIF-4E available for the formation of the eIF-4F complex.

In situations where cap-dependent translation is compromised by cyto- or genotoxic stress, cap-independent protein translation, promoted by internal ribosome entry sites (IRES), is required to maintain expression of critical proteins [12, 13]. This is an alternate mode of translation initiation in which ribosomal subunits are recruited to the IRES by a subset of initiation factors without the participation of eIF-4E. It is thought that IRES-mediated translation is required in eukaryotes for the synthesis of key regulatory proteins in situations where cap-dependent translation is impaired, such as apoptosis or DNA damage [14, 15]. Indeed, it was shown that IRES activity of several mRNAs encoding for proteins involved in cell cycle regulation and apoptosis increases under conditions of cellular stress, which includes DNA damage caused by etoposide treatment [16] or UV irradiation [17].

We and others discovered that an IRES sequence is present in the 5′-untranslated region (UTR) of the p53 mRNA [8, 18]. We also found that the IRES activity of the p53 mRNA increases following DNA damage in MCF-7 cells [4, 8]. MCF-7 is a breast cancer cell line that contains wild-type p53 and has increased synthesis of p53 following DNA damage [8]. This result suggests that this IRES sequence plays a key role in regulating p53 synthesis following DNA damage or other cellular stress.

The presence of an IRES sequence in an isoform of p53, p47 (also known as p53/p47, Δ40p53, and ΔNp53), and a p53 homologue, p73, has also been discovered [18–20]. The increase of p53 IRES activity following genotoxic or other cellular stress was further confirmed by a number of other reports [21–28]. For instance, it was found that during DNA damage or oncogene induced senescence (OIS), the p53 IRES exhibits enhanced activity to facilitate p53 translation [22], which provides further evidence that the p53 IRES plays a key role in regulation of p53 synthesis following DNA damage and OIS. More recently, it was shown that IRES activity of p53 increases in response to glucose deprivation, which links p53 IRES activity with metabolic stress [28].

Control of translational initiation at cellular IRESs requires the presence of auxiliary factors that are known as IRES-*trans* acting factors or ITAFs [12, 29]. ITAFs are proteins that can positively or negatively affect IRES activity [14]. A number of proteins have been identified as binding to the p53 5′-UTR* in vitro* [30]. Many of them are also known to be involved in multiple critical cellular events, including protein translation and ribosomal biogenesis. Therefore, some of these proteins could be potential p53 ITAFs that regulate p53 IRES activity and p53 synthesis. However, to date, there are no reports on whether any of these proteins are potential ITAFs of the p53 IRES. In this study, we discovered two novel, positive regulators of the p53 IRES, translational control protein 80 (TCP80) and RNA helicase A (RHA), from these proteins. Our results also suggest that the interaction between these two proteins is important for the p53 induction and its tumor suppressive function in response to DNA damage.

## 2. Experimental Procedures

### 2.1. Materials

Etoposide was from Calbiochem. The antibodies include anti-DRBP76 (TCP80) antibody (BD Transduction Laboratories), anti-DHX9 (RHA) antibody (Bethyl Laboratories), and anti-*β*-actin antibody (Sigma). The HRP-conjugated p53 antibody for immunoblotting was from Santa Cruz Biotechnology. The original pcDNA3.1/HisB/TCP80 expression vector was from Dr. Michael B. Matthews. TCP80 Δ401-702 and TCP80 Δ640-702 were obtained by regional deletion of the TCP80 vector. Vectors containing altered first or second dsRBM of TCP80 were mutated using the Quick Change site-directed mutagenesis kit from Stratagene. The TCP80 mdsRBM1 vector was obtained by mutating two lysines to glutamate (K450E and K451E) in the first dsRBM, while the TCP80 mdsRBM2 vector was obtained by mutating two lysines to glutamate (K573E and K574E) of the second dsRBM. The pcDNA3.1/RHA expression vector was from Dr. Suisheng Zhang.

### 2.2. Cell Culture and Transfection

MCF-7 and H1299 cells were grown in DMEM medium supplemented with antibiotics and 10% fetal bovine serum (FBS). All plasmid transfections were performed using Fugene 6 transfection reagent (Roche). Cells were seeded in six-well plates and allowed to grow overnight. They were then transfected with 1.5 *μ*g of DNA. Between 24 and 48 hours following transfection, the cells were lysed.

### 2.3. Dual-Luciferase Assays

Cells were lysed with 1x passive lysis buffer (Promega). The Dual-Luciferase Reporter Assay System (Promega) was then used in conjunction with a Berthold luminometer to determine Firefly and Renilla luciferase activities according to manufacturer's instructions.

### 2.4. Immunoprecipitation and RT-PCR

Immunoprecipitation and RT-PCR was performed using the method as previously described [31]. Briefly, MCF-7 cells were lysed in a polysome lysis buffer [31]. Protein G-plus agarose beads (Calbiochem) were coated with an anti-Xpress antibody overnight. The beads were then washed several times and incubated with MCF-7 cell lysate for 2 hours at room temperature. The immunoprecipitated messenger ribonucleoprotein (mRNP) complexes were then washed extensively and treated with proteinase K. The mRNA was extracted using Tri-LS reagent (MRC) and further purified using RNeasy mini columns (Qiagen). The purified RNA was reverse-transcribed using the SuperScript First-Strand synthesis system for RT-PCR (Invitrogen) and the cDNA was amplified using the Expand High Fidelity PCR system (Roche) using primers flanking the p53 IRES (~145 bp). The resulting PCR fragments were run on a 1% agarose gel containing ethidium bromide and visualized using a transilluminator.

### 2.5. Cell Extract Preparation, SDS-PAGE, and Western Blot

Cells were washed twice with phosphate buffered saline and lysed with TGN lysis buffer [8] containing 1% NP-40 and a protease inhibitor cocktail tablet (Roche). Protein concentration was measured using the Lowry assay method. Equal amounts of protein from each cell lysate were loaded onto an SDS-PAGE gel. After electrophoresis, proteins were transferred onto either a nitrocellulose or PVDF membrane.

### 2.6. Coimmunoprecipitation

Subconfluent MCF-7 cells were lysed by TGN lysis buffer and RHA was immunoprecipitated by mixing cell lysate containing equal amounts of protein with an antibody against RHA and protein A/G agarose beads overnight. The mixture was then centrifuged and the precipitated beads were washed three times with TGN lysis buffer followed by addition of SDS sample loading buffer.

## 3. Results

As stated earlier, a previous study has identified multiple proteins that bind to the p53 5′-UTR using an* in vitro* RNA pull-down assay [30]. We wanted to determine whether some of these proteins can act as activators of the p53 IRES to stimulate p53 IRES activity in response to DNA damage. Translational control protein 80 (TCP80), also known as nuclear factor 90 (NF90) or double-stranded RNA binding protein 76 (DRBP76), was one of the proteins that were found to bind to the p53 5′-UTR* in vitro*. It is a double stranded-RNA binding protein [32] and has documented roles in the regulation of protein translation [33]. TCP80 is also involved in IRES-mediated protein translation by acting as an ITAF of the rhinovirus type 2 IRES [34]. Therefore, the ability of TCP80 to associate with p53 IRES* in vivo *was investigated. The binding between TCP80 and p53 IRES in the presence of DNA damage was also assessed.

### 3.1. TCP80 Binds to the p53 mRNA* In Vivo*

**Figure 1:**
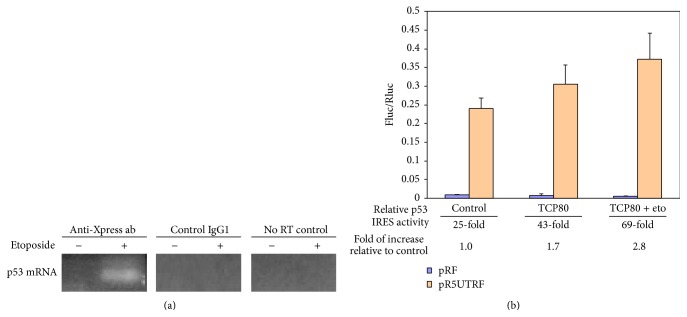
(a) TCP80 has increased binding to the p53 mRNA following DNA damage. MCF-7 cells were transfected with pcDNA3.1/HisB/TCP80 that encodes for the Xpress-tagged TCP80 protein. Twenty-four hours following transfection, the cells were treated with or without 10 *μ*M etoposide for 2 hours. They were then lysed in polysome lysis buffer and incubated with protein G-plus agarose beads coated with the anti-Xpress antibody. The TCP80 and mRNA complexes (mRNP) were immunoprecipitated and mRNA was extracted from the immunoprecipitate as described in Experimental procedures. RT-PCR was then performed to reverse-transcribe and amplify the p53 IRES sequence (~145 bp). (b) TCP80 positively affects the p53 IRES activity in response to DNA damage. MCF-7 cells were cotransfected with pRF or pR5UTRF along with either pcDNA3.1 or pcDNA3.1/HisB/TCP80. Twenty-four hours following the transfection, the cells were treated with or without etoposide for 2 hours. The cells were then lysed and a dual-luciferase assay was performed to detect firefly (Fluc) and renilla (Rluc) luciferase activities as described in Experimental procedures. The results presented are average ± SEM from three individual experiments.

An Xpress-tagged TCP80 protein was overexpressed in MCF-7 cells, and TCP80/mRNA complexes were subsequently immunoprecipitated with the anti-Xpress antibody. RT-PCR was then used to amplify the p53 IRES sequence from the immunoprecipitated TCP80/mRNA complexes. Amplification of the p53 IRES mRNA was observed in the immunoprecipitate derived from MCF-7 cells treated with etoposide, a DNA damage agent that induces DNA double-stranded breaks (Figure 1(a)). In contrast, no amplification was observed in immunoprecipitate obtained from the untreated control samples. These results suggest that TCP80 associates with p53 mRNA* in vivo* and binding of TCP80 to p53 mRNA increases in response to etoposide-induced DNA damage.

### 3.2. TCP80 Upregulates p53 IRES Activity in Response to DNA Damage

TCP80 affects protein translation and has increased binding with p53 mRNA following DNA damage. Therefore, it is conceivable that TCP80 may modulate p53 IRES activity in response to DNA damage. The bicistronic dual-luciferase reporter vector pR5UTRF, which contains the p53 IRES sequence, was used to determine p53 IRES activity in cellular systems [8]. Additionally, the empty vector (pRF) was used as a negative control for pR5UTRF. To determine whether or not TCP80 can affect p53 IRES activity, MCF-7 cells were cotransfected with either pRF or pR5UTRF along with a plasmid expressing TCP80. p53 IRES activity was then measured as the ratio of firefly luciferase (Fluc), which is controlled by the p53 IRES, to renilla luciferase (Rluc) activity [8]. Renilla luciferase is controlled by cap-dependent translational machinery and is used as the internal control. A nearly 2-fold increase in the relative p53 IRES activity was observed in MCF-7 cells overexpressing TCP80 as compared to the control cells (Figure 1(b)). More importantly, when the MCF-7 cells overexpressing TCP80 were also treated with etoposide, a nearly 3-fold increase of relative IRES activity was observed as compared to the control cells (Figure 1(b)). Considering the fact that etoposide treatment alone only leads to nearly 2-fold increase of p53 IRES activity in MCF-7 cells [4, 8], these results indicate that TCP80 not only is a positive modulator of p53 IRES activity but also causes an increase of p53 IRES activity in response to DNA damage.

### 3.3. TCP80 dsRBMs Are Important for the Induction of the p53 IRES Activity

**Figure 2:**
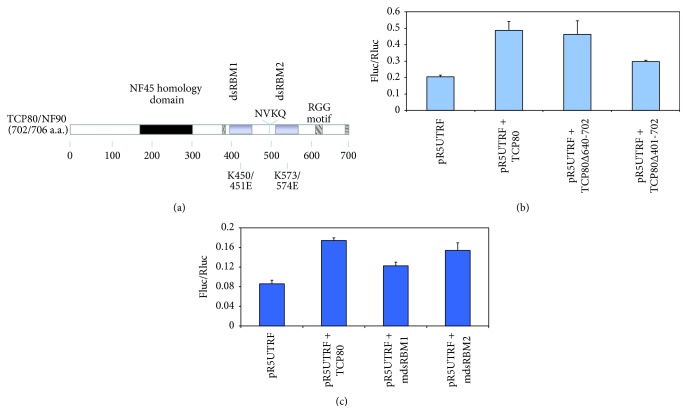
TCP80 dsRBMs are important for the induction of the p53 IRES activity. (a) Map of TCP80/NF90 functional domains. TCP80 contains three RNA binding sites: the RGG domain and two dsRBMs. The mutated residues in the mdsRBM1 and mdsRBM2 constructs, respectively, are also shown in this diagram. (b) Effect of deletion of the RGG domain and the dsRBMs on TCP80-mediated p53 IRES induction. MCF-7 cells were transfected with pR5UTRF along with wild-type TCP80, TCP80Δ640-702 (minus RGG domain), or TCP80Δ401-702 (minus RGG and dsRBMs). Twenty-four hours after transfection, cells were lysed, and a dual-luciferase assay was performed. (c) Effect of mutations in the dsRBMs on TCP80-mediated p53 IRES induction. MCF-7 cells were transfected with pR5UTRF along with plasmids encoding wild-type TCP80, mutant dsRBM1 TCP80 (mdsRBM1), or mutant dsRBM2 TCP80 (mdsRBM2). Firefly and renilla luciferase activities were determined. The results presented in both (b) and (c) are average ± SEM from three individual experiments.

The TCP80 protein contains three RNA binding domains (Figure 2(a)). It has two double-stranded RNA binding motifs (dsRBMs) and one RGG (arginine-glycine-glycine) domain located at its C-terminus [35]. These domains have been known to play an important role in RNA-protein interactions [36]. Therefore, we wanted to determine which of the three domains of TCP80 is important for its interaction with the p53 IRES.

We tested the ability of mutants lacking the TCP80 RGG motif (TCP80 Δ640-702) or the TCP80 RGG motif plus the two dsRBMs (TCP80 Δ401-702) to stimulate p53 IRES activity in MCF-7 cells. We found that the deletion of the RGG domain alone did not affect TCP80's ability to stimulate p53 IRES activity, whereas the additional deletion of both dsRBMs did result in a 40% decrease in p53 IRES stimulation as compared to wild-type TCP80 (Figure 2(b)). These results suggest that the two dsRBMs of TCP80 are important for the interaction between TCP80 and the p53 IRES. We then used plasmids containing mutations in either the first dsRBM (mdsRBM1) or the second dsRBM (mdsRBM2) of TCP80 to determine which of the two dsRBMs is more important for the p53 IRES activity, using full-length TCP80 as the control. These mutants have been known to be important for the interaction between TCP80 and its associated RNAs (Jiang and Miskimins, unpublished observations). Our results revealed that mutations in dsRBM1 led to a 30% decrease in p53 IRES stimulation as compared to wild-type TCP80, but mutations in dsRBM2 led to a 10% decrease in the p53 IRES stimulation as compared to wild-type TCP80 (Figure 2(c)). These results suggest that the first dsRBM is more important for TCP80-induced p53 IRES activation.

### 3.4. TCP80 and RHA Bind to Each Other* In Vivo*

**Figure 3:**
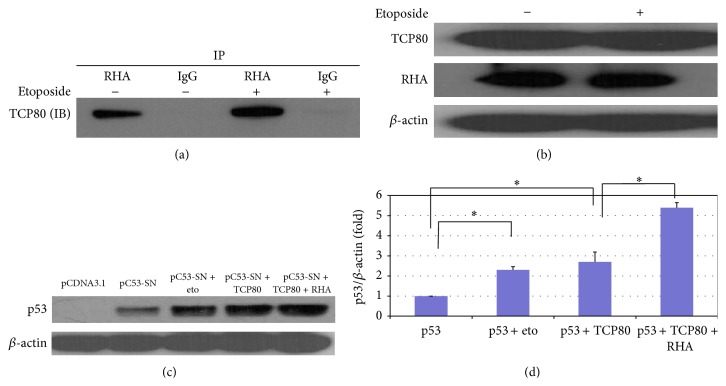
TCP80 and RHA interact* in vivo* and cooperatively stimulate p53 expression in MCF-7 cells. (a) TCP80 has increased binding with RHA following DNA damage in MCF-7 cells. Subconfluent MCF-7 cells were treated with or without 10 *μ*M etoposide for 2 hours and then lysed with TGN buffer [8]. RHA was immunoprecipitated from the cell lysate as described in experimental procedures. The precipitated beads were then washed three times with TGN lysis buffer and SDS sample loading buffer was added. The samples were subjected to SDS-PAGE. An immunoblotting experiment was then performed to detect the TCP80 protein. The results presented are representative of three individual experiments. (b) Levels of TCP80 and RHA protein do not change following exposure to DNA damage in MCF-7 cells. MCF-7 cells were treated with 10 *μ*M etoposide for 2 hours and then lysed with TGN lysis buffer. The samples were subjected to SDS-PAGE. TCP80, RHA, and *β*-actin were detected by their respective antibodies. The results presented in (a) and (b) are representative of three individual experiments. (c) Overexpression of TCP80 and RHA leads to increased p53 expression in H1299 cells transfected with the pC53-SN3 vector. H1299 lung carcinoma cells (p53-null) were cotransfected with the p53 expression vector pC53-SN3 along with the empty pCDNA 3.1 vector, the TCP80 expression vector, or the TCP80 plus RHA expression vector. Twenty-four hours after transfection, the cells were treated with or without etoposide for 2 hours. Cells were then lysed, and equal amounts of protein were subjected to SDS-PAGE and transferred to a nitrocellulose membrane. The p53 protein and *β*-actin were then detected by their respective antibodies. (d) Statistical analysis of the expression levels of p53 (p53/*β*-actin) between individual groups as shown in (c) was performed using one-way ANOVA with a Newman–Keul post hoc test from 4 sets of experimental results. Significance was assumed at ^∗^ *P* < 0.05.

In addition to TCP80, RNA helicase A (RHA) or nuclear DNA helicase II (NDH II) was also identified to bind to the p53 5′-UTR* in vitro* [30]. Interestingly, RHA is known to associate with TCP80* in vitro* [37] and has a known role in the regulation of protein translation as well [38]. We performed an immunoprecipitation experiment to pull down RHA in MCF-7 cells treated with etoposide and look at the binding between RHA and TCP80. Interestingly, we found increased binding of TCP80 to RHA following DNA damage (Figure 3(a)), while levels of both TCP80 and RHA stay the same before and after DNA damage (Figure 3(b)). Since TCP80 also has increased binding to p53 mRNA following DNA damage (Figure 1(a)), these results suggest that the interaction between TCP80 and RHA may be important for p53 IRES activity and p53 induction in response to DNA damage.

### 3.5. RHA Cooperates with TCP80 to Stimulate p53 Expression

Next, we examined the effect of overexpression of TCP80 on levels of p53 in H1299 (p53-null) lung carcinoma cells. Transfection of the pC53-SN3 vector, which contains the p53 IRES sequence (~140 bp) and p53 ORF, in H1299 cells resulted in expression of the p53 protein. When H1299 cells were cotransfected with the pC53-SN3 vector and a plasmid encoding TCP80, a significant increase in p53 levels was observed when compared to cells cotransfected with the pC53-SN3 and the empty vector (Figure 3(c)). The level of increase in p53 expression was similar to that observed in cells transfected with pC53-SN3 and treated with etoposide (Figure 3(c)). Interestingly, when both TCP80 and RHA were overexpressed in H1299 cells transfected with the pC53-SN3 vector, a much greater increase of p53 expression was observed (Figures 3(c) and 3(d)), suggesting a cooperative effect of TCP80 and RHA on p53 expression.

### 3.6. Decreased Expression of TCP80 and RHA in MCF-7 Cells Leads to Diminished p53 Induction following DNA Damage and Decreased Expression of Its Downstream Target PUMA

**Figure 4:**
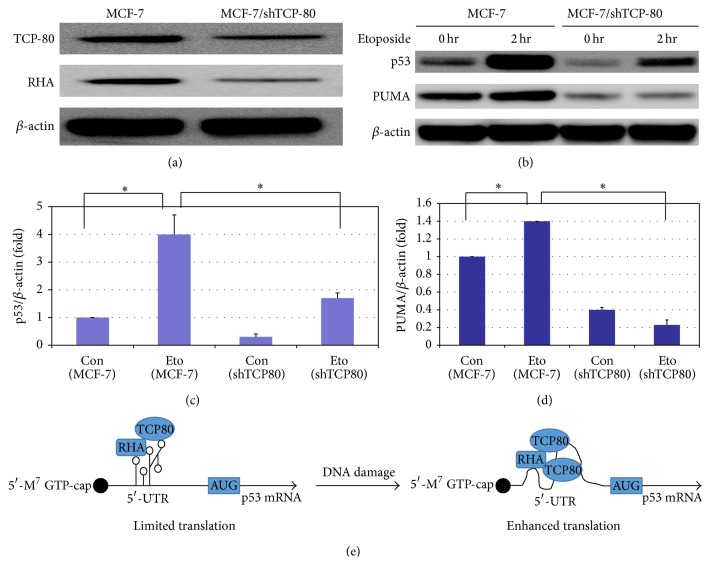
(a) MCF-7/shTCP80 cells express lower levels of TCP80 and RHA as compared to MCF-7 cells. MCF-7 and MCF-7/shTCP80 cells were grown to subconfluency. Cells were then lysed and equal amounts of protein were subjected to SDS-PAGE and western blotting. TCP80, RHA, and *β*-actin were detected by immunoblotting. (b) MCF-7/shTCP80 cells exhibit reduced induction of p53 and its downstream target PUMA following DNA damage. MCF-7 and MCF-7/shTCP80 cells were grown to subconfluency. Cells were then treated with 10 *μ*M etoposide for 2 hours. After the treatment, cells were lysed and equal amounts of protein were subjected to SDS-PAGE and transferred to PVDF membranes. p53, PUMA, and *β*-actin proteins were detected with their respective antibodies. (c) Statistical analysis of the expression levels of p53 (p53/*β*-actin) between individual groups as seen in (b) was carried out using one-way ANOVA with a Newman–Keul post hoc test from 3 sets of experimental results. Significance was assumed at ^∗^ *P* < 0.05. (d) Statistical analysis of the expression levels of PUMA (PUMA/*β*-actin) between individual groups as shown in (b) was performed using one-way ANOVA with a Newman–Keul post hoc test from 3 sets of experimental results. Significance was assumed at ^∗^ *P* < 0.05. (e) A diagram showing proposed regulation of p53 IRES activity by TCP80 and RHA. During the basal conditions, the secondary structure of the p53 IRES is largely stabilized and has limited translational activity due to inadequate interaction between TCP80/RHA and the p53 IRES. Following DNA damage, increased binding of TCP80 to the p53 IRES and enhanced interaction between TCP80/RHA and the p53 IRES facilitate the unwinding of the secondary structure of the p53 IRES, allowing increased translation of the p53 mRNA in response to DNA damage.

To further determine the functional link between positive regulators of p53 IRES, such as TCP80 and RHA, and p53 induction following DNA damage, we created a MCF-7 cell line that is stably transfected with a plasmid containing a shRNA against TCP80. Our results indicate that TCP80 expression is markedly reduced in MCF-7/shTCP80 cells as compared to control MCF-7 cells (Figure 4(a)). Since expression levels of TCP80 and RHA are known to be correlated in various cell lines [35], we tested whether a decrease in TCP80 expression would also result in decreased cellular levels of RHA. Our results showed that this is indeed the case, as the MCF-7/shTCP80 cell line also exhibits reduced expression of RHA (Figure 4(a)).

Next, we examined the expression of p53 in MCF-7/shTCP80 and MCF-7 cells after treating the cells with or without etoposide. We found that p53 expression is reduced in MCF-7/shTCP80 cells as compared to MCF-7 cells. More importantly, we also observed a dramatically decreased p53 induction in MCF-7/shTCP80 cells following etoposide treatment as compared to MCF-7 cells (Figures 4(b) and 4(c)). The p53 upregulated modulator of apoptosis (PUMA) is a proapoptotic protein whose transcription is stimulated by the tumor suppressor p53. Our results show that PUMA expression is reduced in MCF-7/shTCP80 cells as compared to MCF-7 cells. Moreover, while we observed significantly increased expression of PUMA in MCF-7 cells in response to DNA damage, the induction of PUMA following DNA damage is essentially abrogated in MCF-7/shTCP80 cells (Figures 4(b) and 4(d)). As a key regulator of apoptotic process, p53 can induce apoptosis by upregulating the expression of PUMA following DNA damage; our results thus suggest that TCP80 and its binding protein RHA may play important roles in IRES-mediated p53 induction and in regulating p53's tumor suppressive function in response to DNA damage.

## 4. Discussion

TCP80 is known to regulate the translation of the acid beta-glucosidase mRNA by binding to its coding sequence [33]. We found that binding of TCP80 to the p53 mRNA* in vivo* increases following DNA damage. Our results also show that TCP80 is a positive regulator of the p53 IRES and its overexpression enhances the p53 IRES activity following DNA damage. Furthermore, TCP80 stimulates p53 IRES activity in great part through its first double-stranded RNA binding domain (dsRBM), indicating that interaction between TCP80's dsRBM and p53 IRES's secondary structure could be important for increased p53 IRES activity following DNA damage. The involvement of TCP80 and its RBM in cellular IRES-mediated protein translation is further supported by a previous report indicating that TCP80 is an ITAF of the rhinovirus type 2 IRES [34].

RHA plays a crucial role in the translation of some viral and cellular mRNAs that contain a posttranscriptional control element (PCE) within their 5′-UTR [38]. The PCE typically forms a complex secondary structure that hinders 40S ribosomal subunit scanning and efficient translation. However, RHA can bind to the PCE and disrupt or open up its secondary structure by modifying RNA-RNA or RNA-protein interactions. This allows for a more efficient scanning of the 40S ribosomal subunit and translation initiation [38]. The region containing the p53 IRES is predicted to have a strong secondary structure [30]. RHA therefore could exert a positive effect on the p53 IRES by aiding in the unwinding of its secondary structure.

TCP80 and RHA proteins were found to bind to each other* in vitro* [19]. We have further confirmed that these two proteins associate with each other in MCF-7 cells. More interestingly, we observed increased binding of TCP80 to RHA and the p53 mRNA following DNA damage, and overexpression of TCP80, along with RHA, leads to increased expression of p53. Interestingly, expression levels of TCP80 and RHA are correlated in various cell lines [35]. We also found that levels of both TCP80 and RHA are low in MCF-7/shTCP80 cells, which leads to decreased expression of p53 and diminished p53 induction following DNA damage.

Our results suggest that the interaction between TCP80 and RHA is important for the stimulation of p53 IRES activity and p53 induction following DNA damage. It is thought that the dsRBMs of TCP80 are also needed for its interaction with RHA [32, 39]. Although we observed that overexpression of RHA leads to enhanced p53 IRES activity, overexpression of RHA alone cannot lead to a further increase in p53 IRES activity following DNA damage (Figure S1) (see Supplementary Material available online at http://dx.doi.org/10.1155/2015/708158). This result suggests that RHA could be mediating its effect on the p53 IRES activity through its interaction with TCP80. One explanation for the cooperative effect of TCP80 and RHA could be that RHA, as an RNA helicase, utilizes its ability to remodel RNA-RNA or RNA-protein interactions to facilitate increased binding of TCP80 to the p53 IRES following DNA damage, therefore leading to increased p53 IRES activity. Additionally, once more TCP80 are bound to p53 IRES, it could further facilitate the interaction between RHA and the p53 IRES so RHA can help unwind the secondary structure of the p53 IRES (Figure 4(e)).

Our results have also shown that reduced expression of TCP80 and RHA can lead to diminished induction of a p53 downstream target PUMA, a proapoptotic protein, following DNA damage. Since PUMA plays a critical role in p53's ability to induce apoptosis and prevent malignant transformation, this finding suggests that defective IRES-mediated p53 translation is involved in tumorigenesis [4]. The expression of TCP80 is known to be greatly reduced in malignant brain tumors of glial origin, and the subcellular localization of TCP80 is altered in these malignant tumors as well [40]. These results suggest abnormal expression or subcellular localization of TCP80 is linked to malignant transformation of normal cells. In addition, it was found that RHA maps to chromosome band 1q25, which is the site of a major prostate cancer susceptibility locus [41]. RHA also upregulates activity of several other tumor suppressors, such as Werner Syndrome Helicase (WRN), that are involved in DNA repair process through interaction with proteins in the DNA damage foci [42, 43]. Therefore, it is possible that alteration or deletion of this locus may result in abrogated RHA function or expression and prevent induction of the p53 IRES and/or other tumor suppressors, thereby increasing the risk of malignant transformation of prostate tumors. The roles of TCP80 and RHA in regulating p53 IRES activity and their involvement in oncogenesis require further investigation.

## 5. Conclusions

To date, the majority of research on p53 and oncogenesis has been aimed at characterizing the genetic mutations or posttranslational modifications that alter the p53 protein and lead to the loss of its transcriptional activity or induction in cancer cells [1, 44]. The mechanisms underlying translational regulation of the p53 tumor suppressor and the role of p53 translation in the prevention of tumorigenesis are significantly understudied. Our discovery of the function of TCP80 and RHA in regulating p53 IRES and p53 induction following DNA damage has provided a better understanding of the mechanisms that regulate IRES-mediated p53 translation in response to genotoxic stress. Given the importance of p53 in preventing tumorigenesis, the results obtained from this study may also provide important insights regarding defective IRES-mediated p53 translation in the pathogenesis of cancer.

## Supplementary Material

Description of Results: We observed that overexpression of RHA leads to enhanced p53 IRES activity under normal growth conditions (Figure S1A). However, in contrast to overexpression of TCP80 (Fig. 1B), overexpression of RHA cannot lead to a further increase in p53 IRES activity following DNA damage (Figure S1B).
