# Supplementary material for: Translational Control Protein 80 Stimulates IRES-Mediated Translation of p53 mRNA in Response to DNA Damage
Source: Biomed Res Int. 2015 Jul 26;2015:708158. doi: 10.1155/2015/708158 (PMC4529924; doi:10.1155/2015/708158)
Supplement: Supplementary file 1 — Description of Results: We observed that overexpression of RHA leads to enhanced p53 IRES activity under normal growth conditions (Figure S1A). However, in contrast to overexpression of TCP80 (Fig. 1B), overexpression of RHA cannot lead to a further increase in p53 IRES activity following DNA damage (Figure S1B). [file 708158.f1.pdf]

Supplemental Materials:

Figure S1. (A) Overexperssion of RHA leads to enhanced p53 IRES activity. MCF-7 cells were co-transfected with pRF or pR5UTRF along with either pcDNA3.1 or pcDNA3.1/HisB/TCP80. Twenty-four hours following the transfection, the cells were lysed and a dual-luciferase assay was performed to detect firefly (Fluc) and renilla (Rluc) luciferase activities as described in Experimental procedures. **(B) Overexperssion of RHA does not lead to a further increase in p53 IRES activity following DNA damage.** MCF-7 cells were co-transfected with pRF or pR5UTRF along with either pcDNA3.1 or pcDNA3.1/HisB/TCP80. Twenty-two hours following the transfection, the cells were treated with etoposide for 2 hours. The cells were then lysed and a dual-luciferase assay was performed to detect firefly (Fluc) and renilla (Rluc) luciferase activities as described above. The results presented in A and B are average ± SEM from three individual experiments.

Figure S1

A.

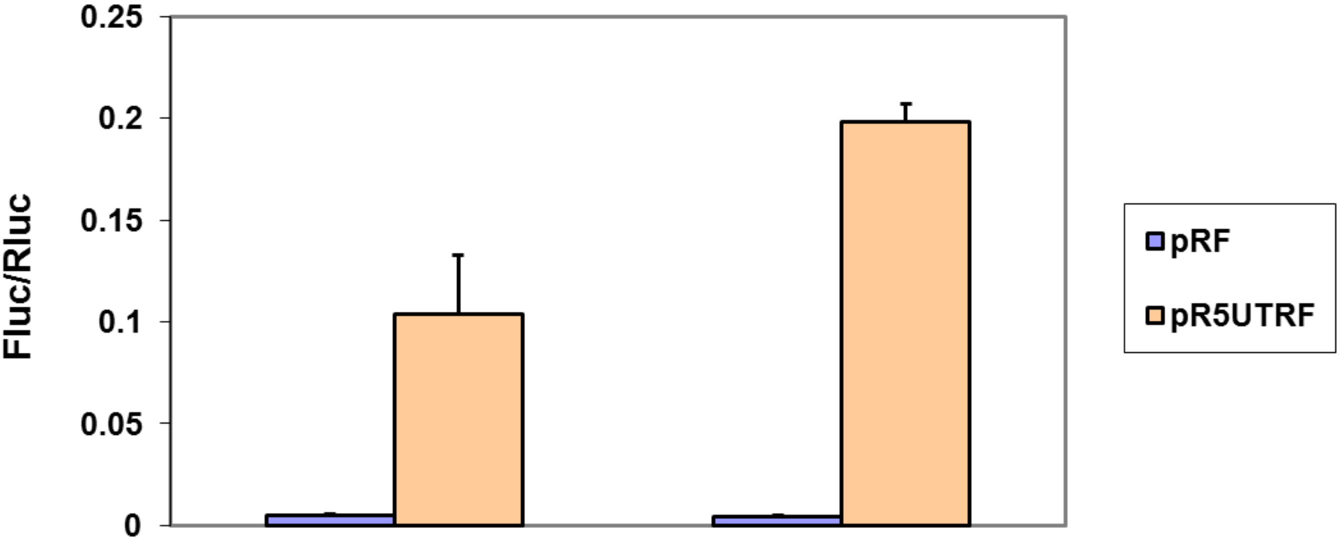

	Control	RHA
Relative p53 IRES activity	20-fold	45-fold
Fold of increase relative to control	1.0	2.2

B.

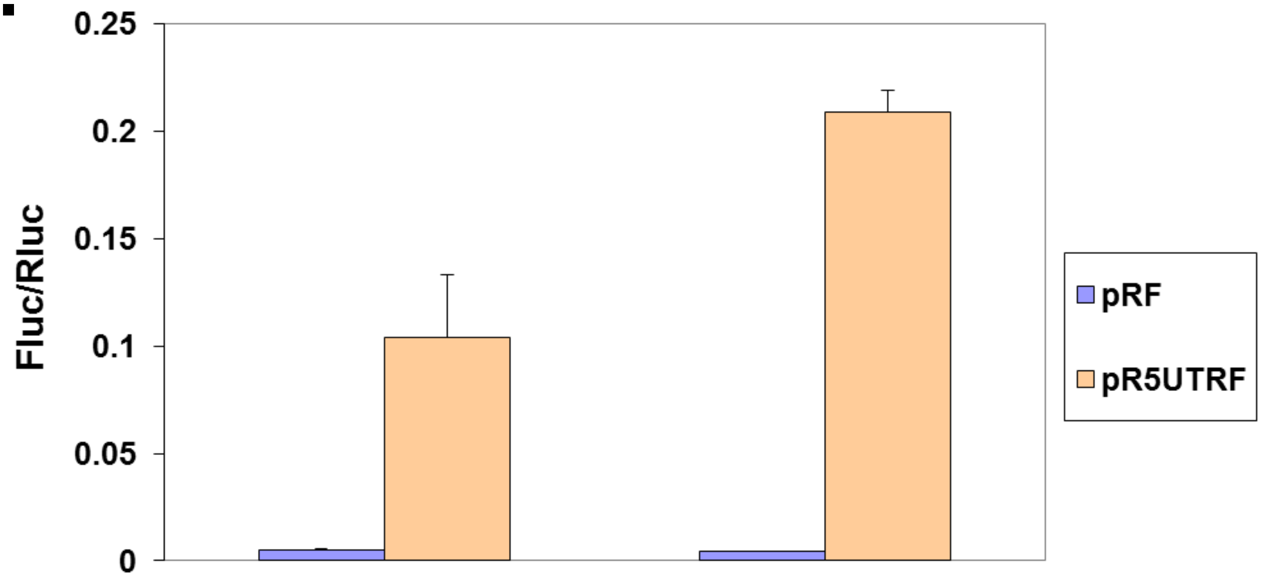

	Control	RHA+eto
Relative p53 IRES activity	20-fold	47-fold
Fold of increase relative to control	1.0	2.3
